# 4-Cyano-1-methyl­pyridinium nitrate

**DOI:** 10.1107/S1600536813014025

**Published:** 2013-05-31

**Authors:** Cameron A. McCormick, Vu D. Nguyen, Heather E. Renfro, Lynn V. Koplitz, Joel T. Mague

**Affiliations:** a Department of Physics, Loyola University, New Orleans, LA 70118, USA; b Department of Chemistry, Loyola University, New Orleans, LA 70118, USA; c Department of Chemistry, Tulane University, New Orleans, LA 70118, USA

## Abstract

The title mol­ecular salt, C_7_H_7_N_2_^+^·NO_3_^−^, displays an inter­penetrating sheet structure parallel to *a* with each sheet containing nearly coplanar cations and anions, each ion being bis­ected by a crystallographic mirror plane. C—H⋯O hydrogen bonds involving both ring and methyl H atoms in addition to cation–cation C—H⋯N hydrogen bonds (ring H to cyano N) serve to link the sheets together. In each set of parallel layers, the cations and anions stack with short distances of 3.094 (2) (between aligned nitrate N and pyridine N atoms) and 3.057 (2) Å (between a nitrate O atom and the ring centroid). This motif is strikingly similar to the one that features in the isomeric salt 2-cyano-1-methyl­pyridinium nitrate.

## Related literature

For structures of other 4-cyano-1-methyl­pyridinium salts, see: Bockman & Kochi (1989 ▶); Bockman & Kochi (1992 ▶); Hardacre *et al.* (2008 ▶, 2010 ▶); Kammer *et al.* (2012*a* ▶,*b* ▶. For the structure of 2-cyano-1-methyl­pyridinium nitrate, see: Koplitz *et al.* (2012 ▶), of 3-cyano-1-methyl­pyridinium chloride, see: Koplitz *et al.* (2003 ▶) and of 3-cyano-*N*-methyl­pyridinium bromide, see: Mague *et al.* (2005 ▶). For a discussion of anion–π inter­actions, see: Frontera *et al.* (2011 ▶). For the structure of 2-cyano­anilinium nitrate, see: Cui & Wen (2008 ▶) and of 3-cyano­anilinium nitrate, see: Wang (2009 ▶).
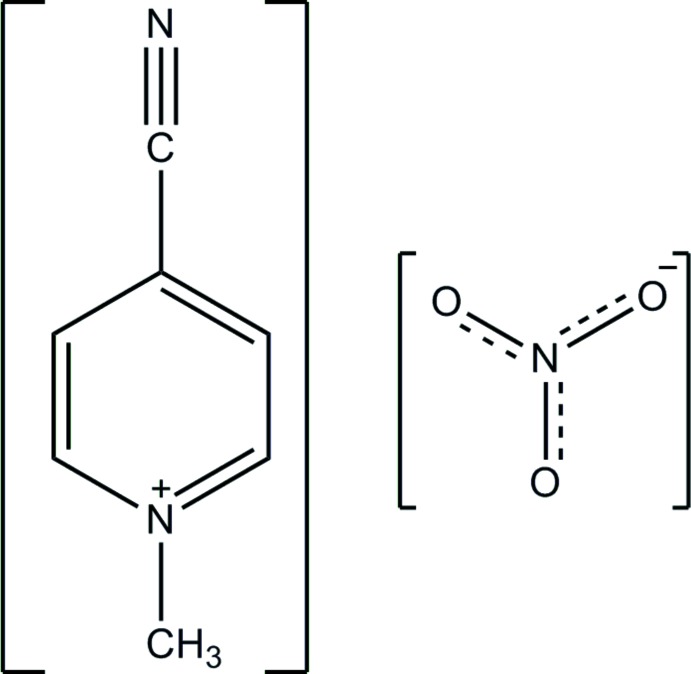


## Experimental

### 

#### Crystal data

C_7_H_7_N_2_^+^·NO_3_^−^
*M* *_r_* = 181.16
Orthorhombic, 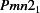

*a* = 8.195 (3) Å
*b* = 7.289 (3) Å
*c* = 6.721 (3) Å
*V* = 401.5 (3) Å^3^
*Z* = 2
Mo *K*α radiation
μ = 0.12 mm^−1^
*T* = 100 K
0.33 × 0.23 × 0.13 mm

#### Data collection

Bruker SMART APEX CCD diffractometer
Absorption correction: multi-scan (*TWINABS*; Sheldrick, 2009 ▶) *T*_min_ = 0.860, *T*_max_ = 0.985
6751 measured reflections
1116 independent reflections
1089 reflections with *I* > 2σ(*I*)
*R*_int_ = 0.091

#### Refinement

*R*[*F* ^2^ > 2σ(*F* ^2^)] = 0.038
*wR*(*F* ^2^) = 0.092
*S* = 1.09
1116 reflections
71 parameters
1 restraint
H-atom parameters constrained
Δρ_max_ = 0.40 e Å^−3^
Δρ_min_ = −0.43 e Å^−3^

### 

Data collection: *APEX2* (Bruker, 2010 ▶); cell refinement: *SAINT* (Bruker, 2009 ▶); data reduction: *SAINT*; program(s) used to solve structure: FLIPPER option in *PLATON* (Spek, 2009 ▶); program(s) used to refine structure: *SHELXL97* (Sheldrick, 2008 ▶); molecular graphics: *DIAMOND* (Brandenburg & Putz, 2012 ▶); software used to prepare material for publication: *SHELXTL* (Sheldrick, 2008 ▶).

## Supplementary Material

Crystal structure: contains datablock(s) I, global. DOI: 10.1107/S1600536813014025/hb7076sup1.cif

Structure factors: contains datablock(s) I. DOI: 10.1107/S1600536813014025/hb7076Isup2.hkl

Supplementary material file. DOI: 10.1107/S1600536813014025/hb7076Isup3.cml

Additional supplementary materials:  crystallographic information; 3D view; checkCIF report

## Figures and Tables

**Table 1:** Hydrogen-bond geometry (Å, °)

*D*—H⋯*A*	*D*—H	H⋯*A*	*D*⋯*A*	*D*—H⋯*A*
C1—H1*A*⋯O1^i^	0.96	2.71	3.3826 (19)	127
C1—H1*A*⋯O1^ii^	0.96	2.71	3.3826 (19)	127
C1—H1*B*⋯O1^iii^	0.90	2.60	3.4485 (15)	159
C2—H2⋯O1^iv^	0.95	2.65	3.3763 (17)	134
C2—H2⋯O2^iv^	0.95	2.29	3.2379 (15)	172
C3—H3⋯N2^iv^	0.95	2.51	3.2272 (15)	132
C3—H3⋯O1^v^	0.95	2.56	3.2568 (17)	131

Symmetry codes: (i) 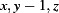; (ii) 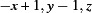; (iii) 
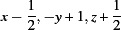
; (iv) 
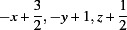
; (v) 
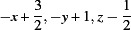
.

**Fig. 1.**
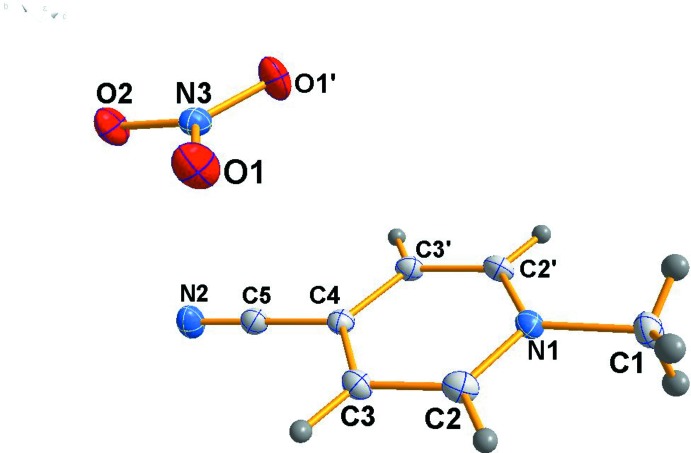
Perspective view of I with displacement ellipsoids drawn at the 50% probability level and H-atoms as spheres of arbitrary radius. Primed atoms are related to unprimed counterparts by 1-x, y, z.

**Fig. 2.**
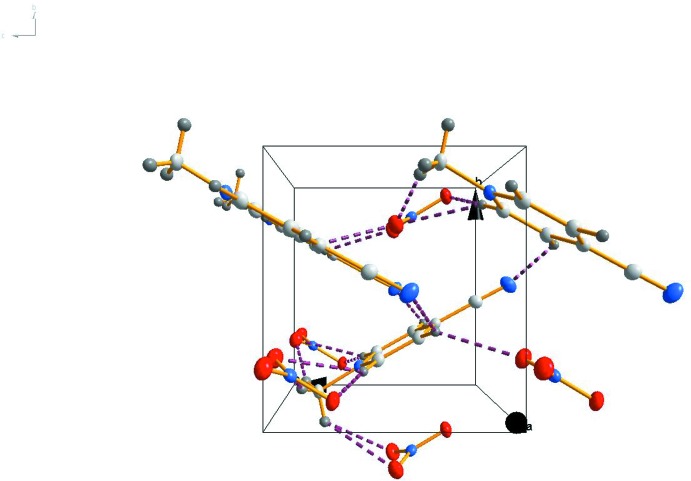
Packing of I viewed down a showing the interpenetrating layers. The C—H···O and C—H···N interactions are shown as dashed lines.

**Fig. 3.**
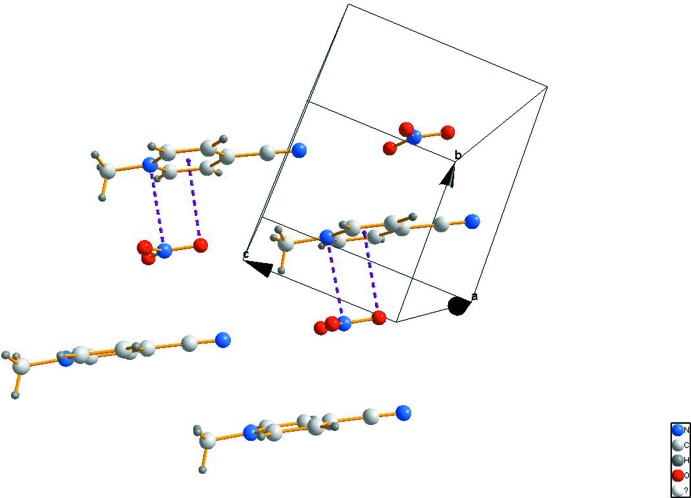
Packing of I showing the anion–π interactions as dashed lines.

## supplementary crystallographic information

### Comment

A perspective view of the title compound appears in Fig. 1 while Fig. 2 illustrates the interpenetrating sets of parallel cation/anion sheets. Within each layer, the dihedral angle between mean cation and anion planes is 1.63 (3)° while the two sets of layers are inclined at an angle of 60.05 (4)°. The majority of the interionic interactions are C—H···O hydrogen bonds between cations in one set of layers and anions in the other set. Additionally there are C—H···N interactions between ring H atoms of cations in one set of layers and the cyano groups of cations in the other set (Table 1 and Fig. 3). A notable feature is the close interlayer cation-anion contact which is strikingly similar to the motif that dominates the structure of 2-cyano-1-methylpyridinium nitrate. (Koplitz *et al.*, 2012). Thus, the N3—O2 bond of one anion is oriented with O2 lying directly over the centroid of the nearest parallel pyridinium ring at a distance of 3.057 (2) Å and N3 lying directly over the pyridinium nitrogen (N1) at a distance of 3.094 (2) Å. These close contacts are likely the result of electrostatic cation-anion attraction with the orientation possibly reinforced by an anion-π interaction (Frontera *et al.*, 2011). In contrast to the structure found for the title compound, the structures of the isomeric salts 2-cyano-1-methylpyridinium nitrate (Koplitz *et al.*, 2012) and 2-cyanoanilinium nitrate (Cui & Wen, 2008) crystallize in flat layers of two-dimensional networks with only a few atoms protruding from the mirror plane while 3-cyanoanilinium nitrate (Wang, 2009) forms a more open structure.

### Experimental

4-Cyanopyridine (10.55 g) was dissolved in benzene (40 ml). Iodomethane (9.5 ml) was added to this solution slowly with stirring and the solution was refluxed for 75 minutes. Yellow solid 4-cyano-*N*-methylpyridinium iodide (m.p. 189–193° C) was collected by vacuum filtration. This solid (0.226 g) was then dissolved in ethanol (20.3 ml) along with an equimolar amount lead(II) nitrate (0.1487 g). Precipitated PbI_2_ was removed by vacuum filtration and the filtrate containing 4-cyano-*N*-methylpyridinium nitrate was slowly evaporated to dryness to form colourless blocks of the title compound.

### Refinement

H-atoms were placed in calculated positions (C—H = 0.95 - 0.98 Å) and included as riding contributions with isotropic displacement parameters 1.2 - 1.5 times those of the attached carbon atoms. Because both ions sit on the mirror plane, the methyl group H atoms are disordered across the mirror. Trial refinements with both the one-component reflection file extracted from the full data set with TWINABS and with the full two-component file showed that use of the former provided a better refinement.

### Crystal data

**Table:**

C_7_H_7_N_2_^+^·NO_3_^−^	*F*(000) = 188
*M**_r_* = 181.16	*D*_x_ = 1.499 Mg m^−^^3^
Orthorhombic, *P**m**n*2_1_	Mo *K*α radiation, λ = 0.71073 Å
Hall symbol: P 2ac -2	Cell parameters from 5849 reflections
*a* = 8.195 (3) Å	θ = 2.8–29.1°
*b* = 7.289 (3) Å	µ = 0.12 mm^−^^1^
*c* = 6.721 (3) Å	*T* = 100 K
*V* = 401.5 (3) Å^3^	Block, colourless
*Z* = 2	0.33 × 0.23 × 0.13 mm

### Data collection

**Table:**

Bruker SMART APEX CCD diffractometer	1116 independent reflections
Radiation source: fine-focus sealed tube	1089 reflections with *I* > 2σ(*I*)
Graphite monochromator	*R*_int_ = 0.091
φ and ω scans	θ_max_ = 29.1°, θ_min_ = 2.8°
Absorption correction: multi-scan (*TWINABS*; Sheldrick, 2009)	*h* = −11→11
*T*_min_ = 0.860, *T*_max_ = 0.985	*k* = −9→9
6751 measured reflections	*l* = −9→8

### Refinement

**Table:**

Refinement on *F*^2^	Secondary atom site location: difference Fourier map
Least-squares matrix: full	Hydrogen site location: inferred from neighbouring sites
*R*[*F*^2^ > 2σ(*F*^2^)] = 0.038	H-atom parameters constrained
*wR*(*F*^2^) = 0.092	*w* = 1/[σ^2^(*F*_o_^2^) + (0.0602*P*)^2^ + 0.031*P*] where *P* = (*F*_o_^2^ + 2*F*_c_^2^)/3
*S* = 1.09	(Δ/σ)_max_ < 0.001
1116 reflections	Δρ_max_ = 0.40 e Å^−^^3^
71 parameters	Δρ_min_ = −0.43 e Å^−^^3^
1 restraint	Extinction correction: *SHELXL* (Sheldrick, 2008), Fc^*^=kFc[1+0.001xFc^2^λ^3^/sin(2θ)]^-1/4^
Primary atom site location: structure-invariant direct methods	Extinction coefficient: 0.098 (15)

### Special details

**Table:**

Experimental. The diffraction data were obtained from 3 sets of 400 frames, each of width 0.5° in ω, collected at φ = 0.00, 90.00 and 180.00° and 2 sets of 800 frames, each of width 0.45\5 in φ, collected at ω = -30.00 and 210.00°. The scan time was 15 sec/frame. Analysis of 427 reflections chosen from the full data set and having I/σ(I) > 15.0 with *CELL_NOW* (Sheldrick, 2008*a*) showed the crystal to belong to the orthorhombic system and to be twinned by a 180° rotation about *c*. The raw data were processed with *SAINT* under control of the 2-component orientation file generated by *CELL_NOW*.
Geometry. All e.s.d.'s (except the e.s.d. in the dihedral angle between two l.s. planes) are estimated using the full covariance matrix. The cell e.s.d.'s are taken into account individually in the estimation of e.s.d.'s in distances, angles and torsion angles; correlations between e.s.d.'s in cell parameters are only used when they are defined by crystal symmetry. An approximate (isotropic) treatment of cell e.s.d.'s is used for estimating e.s.d.'s involving l.s. planes.
Refinement. Refinement of *F*^2^ against ALL reflections. The weighted *R*-factor *wR* and goodness of fit *S* are based on *F*^2^, conventional *R*-factors *R* are based on *F*, with *F* set to zero for negative *F*^2^. The threshold expression of *F*^2^ > σ(*F*^2^) is used only for calculating *R*-factors(gt) *etc*. and is not relevant to the choice of reflections for refinement. *R*-factors based on *F*^2^ are statistically about twice as large as those based on *F*, and *R*- factors based on ALL data will be even larger. H-atoms were placed in positions derived from a difference map and their coordinates adjusted to give C—H = 0.95 Å (aromatic) and 0.98 Å (aliphatic). All were included as riding contributions with isotropic displacement parameters 1.2 - 1.5 times those of the attached carbon atoms.

### Fractional atomic coordinates and isotropic or equivalent isotropic displacement parameters (Å^2^)

**Table:**

	*x*	*y*	*z*	*U*_iso_*/*U*_eq_	
N1	0.5000	0.16216 (16)	0.63902 (16)	0.0149 (3)	
N2	0.5000	0.51281 (19)	−0.0553 (2)	0.0246 (3)	
C1	0.5000	0.0548 (2)	0.8274 (2)	0.0191 (3)	
H1A	0.5000	−0.0746	0.7980	0.029*	
H1B	0.4089	0.0825	0.8949	0.029*	
C2	0.64416 (13)	0.20717 (15)	0.55473 (14)	0.0168 (2)	
H2	0.7434	0.1753	0.6192	0.020*	
C3	0.64799 (13)	0.29942 (14)	0.37491 (15)	0.0163 (2)	
H3	0.7489	0.3309	0.3142	0.020*	
C4	0.5000	0.34509 (18)	0.2849 (2)	0.0146 (3)	
C5	0.5000	0.4394 (2)	0.0962 (2)	0.0175 (3)	
N3	0.5000	0.80142 (16)	0.39635 (19)	0.0154 (3)	
O1	0.63291 (10)	0.75855 (13)	0.47555 (13)	0.0244 (2)	
O2	0.5000	0.89102 (15)	0.23498 (16)	0.0202 (3)	

### Atomic displacement parameters (Å^2^)

**Table:**

	*U*^11^	*U*^22^	*U*^33^	*U*^12^	*U*^13^	*U*^23^
N1	0.0165 (7)	0.0168 (5)	0.0114 (6)	0.000	0.000	−0.0014 (5)
N2	0.0183 (7)	0.0289 (7)	0.0267 (6)	0.000	0.000	0.0087 (6)
C1	0.0233 (9)	0.0218 (7)	0.0121 (6)	0.000	0.000	0.0014 (5)
C2	0.0139 (5)	0.0200 (5)	0.0165 (5)	−0.0001 (3)	−0.0018 (4)	−0.0028 (4)
C3	0.0136 (5)	0.0189 (4)	0.0164 (5)	−0.0016 (4)	0.0010 (4)	−0.0009 (4)
C4	0.0161 (7)	0.0140 (6)	0.0138 (7)	0.000	0.000	−0.0018 (5)
C5	0.0134 (7)	0.0183 (6)	0.0209 (7)	0.000	0.000	0.0011 (5)
N3	0.0177 (7)	0.0144 (5)	0.0140 (6)	0.000	0.000	−0.0026 (5)
O1	0.0179 (4)	0.0329 (4)	0.0224 (4)	0.0042 (3)	−0.0036 (3)	0.0043 (3)
O2	0.0205 (6)	0.0265 (5)	0.0138 (5)	0.000	0.000	0.0035 (4)

### Geometric parameters (Å, º)

**Table:**

N1—C2^i^	1.3506 (12)	C3—C4	1.3955 (13)
N1—C2	1.3506 (12)	C3—H3	0.9500
N1—C1	1.4887 (18)	C4—C3^i^	1.3955 (13)
N2—C5	1.150 (2)	C4—C5	1.443 (2)
C1—H1A	0.9638	N3—O1^i^	1.2519 (11)
C1—H1B	0.8964	N3—O1	1.2520 (11)
C2—C3	1.3834 (15)	N3—O2	1.2660 (17)
C2—H2	0.9500		
			
C2^i^—N1—C2	122.01 (12)	C2—C3—H3	120.8
C2^i^—N1—C1	118.98 (6)	C4—C3—H3	120.8
C2—N1—C1	118.98 (6)	C3^i^—C4—C3	120.70 (13)
N1—C1—H1A	109.9	C3^i^—C4—C5	119.65 (7)
N1—C1—H1B	108.2	C3—C4—C5	119.65 (7)
H1A—C1—H1B	108.9	N2—C5—C4	179.23 (15)
N1—C2—C3	120.29 (10)	O1^i^—N3—O1	120.92 (13)
N1—C2—H2	119.9	O1^i^—N3—O2	119.54 (6)
C3—C2—H2	119.9	O1—N3—O2	119.54 (6)
C2—C3—C4	118.35 (10)		
			
C2^i^—N1—C2—C3	−1.14 (19)	C2—C3—C4—C3^i^	0.29 (18)
C1—N1—C2—C3	176.98 (10)	C2—C3—C4—C5	−179.29 (11)
N1—C2—C3—C4	0.41 (15)		

Symmetry code: (i) −*x*+1, *y*, *z*.

### Hydrogen-bond geometry (Å, º)

**Table:**

*D*—H···*A*	*D*—H	H···*A*	*D*···*A*	*D*—H···*A*
C1—H1*A*···O1^ii^	0.96	2.71	3.3826 (19)	127
C1—H1*A*···O1^iii^	0.96	2.71	3.3826 (19)	127
C1—H1*B*···O1^iv^	0.90	2.60	3.4485 (15)	159
C2—H2···O1^v^	0.95	2.65	3.3763 (17)	134
C2—H2···O2^v^	0.95	2.29	3.2379 (15)	172
C3—H3···N2^v^	0.95	2.51	3.2272 (15)	132
C3—H3···O1^vi^	0.95	2.56	3.2568 (17)	131

Symmetry codes: (ii) *x*, *y*−1, *z*; (iii) −*x*+1, *y*−1, *z*; (iv) *x*−1/2, −*y*+1, *z*+1/2; (v) −*x*+3/2, −*y*+1, *z*+1/2; (vi) −*x*+3/2, −*y*+1, *z*−1/2.
